# The Role of Sphingolipids Metabolism in Cancer Drug Resistance

**DOI:** 10.3389/fonc.2021.807636

**Published:** 2021-12-23

**Authors:** Marina Bataller, Almudena Sánchez-García, Yoelsis Garcia-Mayea, Cristina Mir, Isabel Rodriguez, Matilde Esther LLeonart

**Affiliations:** ^1^ Biomedical Research in Cancer Stem Cells Group, Vall d´Hebron Research Institute (VHIR), Barcelona, Spain; ^2^ Assistant Director of Nursing, Nursing Management Service Hospital Vall d’Hebron, Barcelona, Spain; ^3^ Spanish Biomedical Research Network Centre in Oncology, CIBERONC, Madrid, Spain

**Keywords:** glucosylceramide synthase (GCS), sphingosine kinase 1 (SPHK1), sphingomyelinase (SMase), acid ceramidase (AC), cancer, shingolipids

## Abstract

Drug resistance continues to be one of the major challenges to cure cancer. As research in this field evolves, it has been proposed that numerous bioactive molecules might be involved in the resistance of cancer cells to certain chemotherapeutics. One well-known group of lipids that play a major role in drug resistance are the sphingolipids. Sphingolipids are essential components of the lipid raft domains of the plasma membrane and this structural function is important for apoptosis and/or cell proliferation. Dysregulation of sphingolipids, including ceramide, sphingomyelin or sphingosine 1-phosphate, has been linked to drug resistance in different types of cancer, including breast, melanoma or colon cancer. Sphingolipid metabolism is complex, involving several lipid catabolism with the participation of key enzymes such as glucosylceramide synthase (GCS) and sphingosine kinase 1 (SPHK1). With an overview of the latest available data on this topic and its implications in cancer therapy, this review focuses on the main enzymes implicated in sphingolipids metabolism and their intermediate metabolites involved in cancer drug resistance.

## 1 Introduction

Cancer incidence and mortality are growing fast worldwide, with a higher frequency in countries with higher socioeconomic development, as life expectancy continues to rise. However, in countries with stronger health care systems, cancer mortality is decreasing due to early detection and treatment. For several decades, cancer has been the second leading cause of death globally where lung cancer is at the top of the list as the leading cause of cancer deaths. Nevertheless, the most commonly diagnosed types of cancer vary among countries depending on the degree of socioeconomic development and life style factors (1).

Despite the rapid advance of cancer therapies, treatment-resistant relapse remains a major challenge in cancer treatment. Treatment resistance can be classified as intrinsic or acquired resistance, depending on its origin. Intrinsic resistance arises from the administration of chemotherapy treatment, and it is due to preexisting factors of the tumor, so the tumor cannot respond to the initial treatment. On the other hand, acquired resistance appears during or after the administration of treatment and is usually the main contributing factor for relapse. Some theories explain this resistance as sporadic genetic mutations maintained by Darwinian selection through the exposure to the chemotherapeutic agent (2, 3). There are several mechanisms by which tumoral cells acquire this resistance to treatment: inactivation of the drug, multi-drug resistance (MDR) mechanisms, cell death inhibition increasing resistance to apoptosis, changes in cell metabolism, epigenetics modulation, increased DNA repair and gene mutation or amplification that cause the resistance to the chemotherapy (4).

Sphingolipids were named by Thudichum JL in 1884 because of their enigmatic nature (5). It is now known that sphingolipids are a family of bioactive membrane lipids that contribute to the regulation of the fluidity of the plasmatic membrane. The sub-domain structure of the lipid bilayers forms lipid rafts, which act as first and/or second messengers in different pathways as they function as bio-effector molecules (6). The enzymes involved in the sphingolipids’ metabolism have been studied during the last decade and have been directly linked to the control of cell growth, proliferation and apoptosis, among other cellular functions. **Table 1** summarizes the functions and characteristics of the main enzymes of sphingolipids’ metabolism involved in cancer. This review focuses on the importance of these enzymes with a specific focus on their response to drug therapy.

**Table 1:** Functions and characteristics of the main enzymes in sphingolipids’ metabolism involved in cancer.

Enzyme	Cancer	Characteristics and functions		Reference
*Acid ceramidase*	Melanoma	Modulates transition from proliferative to invasive phenotype ↑AC in proliferative melanoma cells ↓AC sensitizes cells to doxorubicin and dacarbazine		(7–10)
	Prostate	↑AC in 60% of prostate cancers ↓AC sensitizes cells to doxorubicin, etoposide, cisplatin and gemcitabine		(11, 12)
	Glioblastoma	↑AC in radioresistant tumors ↑AC increases survival of GSCs		(13, 14)
	HNSCC	↓AC sensitizes cells to FasL gene therapy		(15)
	Breast	Implicated in resistance		(16, 17)
	AML	Induces apoptosis		(18, 19)
*Sphingomyelinases*	Glioblastoma	↑aSMase sensitizes cells to gemcitabine and doxorubicin ↑aSMase does not sensitize cells to temozolomide		(20, 21)
	Melanoma	↓aSMase increases resistance to cisplatin ↑aSMase sensitizes cells to radiotherapy		(22, 23)
	Colon	Cisplatin translocates aSMase and induces apoptosis		(24)
	Ovarian	Cisplatin translocates aSMase and induces apoptosis ↓aSMase increases resistance to paclitaxel		(25, 26)
	NSCLC	Dysfunctional activity in cisplatin-resistant cells		(27)
*Glycosyl ceramide* *synthases*	Breast	↑GCS in adriamycin-resistant cells ↑GCS increases proliferation		(28, 29)
	Colon	↓GCS sensitizes cells to temozolomide		(30)
	Glioblastoma	↓GCS sensitizes cells to paclitaxel		(31)
*Sphingosine kinase*	Prostate	↑SPHK1 in chemoresistant cells ↓SPHK1 sensitizes to camptothecin and docetaxel		(32, 33)
	Breast	ERpositive subtype	Increases proliferation and survival ↑SPHK1 promotes endocrine resistance ↑SPHK1 in doxorubicinresistant cells	(34–37)
		ERnegative subtype	↓SPHK2 sensitizes cells to doxorubicin and etoposide	(38)
	Glioblastoma	↑SPHK1 in glioblastoma cells ↓SPHK1 sensitizes cells to temozolomide		(39–42)
	CML	↑SPHK1 increases resistance to imatinib through PP2A		(43, 44)

## 2 Cellular Functions of Sphingolipids

**Figure 1:**
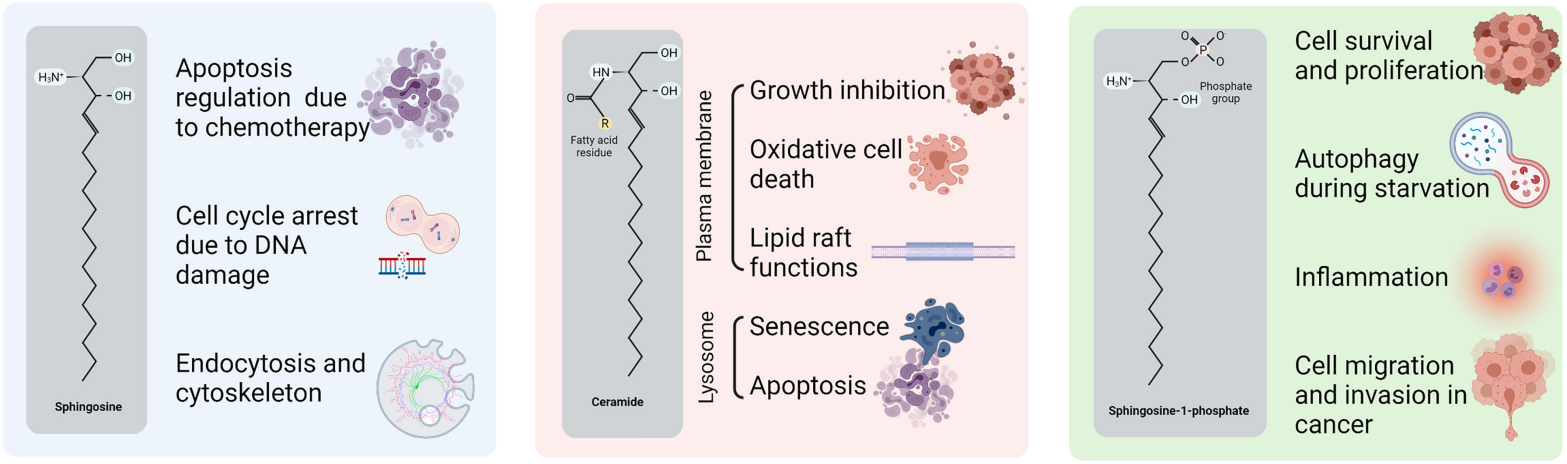
Structure and functions of sphingolipids in cancer. Sphingosine, ceramide and S1P structures with the principal carbon chain and the main chemist groups highlighted. Ceramide functions are divided by the location in the cell.

The first sphingolipid that was identified was sphingosine, whose involvement has been described in the cytoskeleton, endocytosis, cell cycle and apoptosis regulation (45). However, the sphingolipids that have been most frequently implicated in cancer are ceramide and sphingosine 1-phosphate (S1P). Ceramide functions in the cell differ depending on the subcellular location where ceramide is accumulated. For example, when ceramide is generated in the plasma membrane, it is involved in growth inhibition, oxidative stress-mediated cell death, and lipid raft functions (46, 47); whereas when it is located in the lysosomes, ceramide mediates cell-stress responses such as cell senescence and apoptosis (48–50). In contrast, S1P plays a role in cell survival and proliferation, cell migration and invasion, autophagy and inflammation (51, 52). Furthermore, glucosylceramide, a derivative from ceramide, regulates the post-Golgi trafficking, as the enzyme that catalyzes the step from ceramide to glucosylceramide located in the Golgi apparatus (53). **Figure 1** represents the structure of these sphingolipids and some of the main cellular processes in which they are involved in cancer.

## 3 Sphingolipids’ Metabolism

It has been reported that sphingolipids metabolism has a unique metabolic entry point and a unique metabolic exit point. The first one is through the enzyme SPT (serine palmitoyl transferase), which forms the sphingolipid ceramide by the condensation of serine and palmitate in the synthesis pathway. The exit point is mediated by S1P lyase (SGPL), an enzyme that breaks down S1P into different non-sphingolipid molecules. Ceramide has a central position in both the catabolism and anabolism of sphingolipids. For this reason, it is considered as a metabolic hub in the sphingolipids’ metabolism pathway.

Specifically, the synthesis of ceramide starts with the condensation of serine and palmitoyl CoA by SPT, resulting in 3-keto-dihydrosphingosine, which then results in dihydrosphingosine by the action of 3-ketosphinganine reductase (KDSR) and it is later acetylated into dihydroceramide by ceramide synthases (CerS 1-6). Finally, the desaturation of dihydroceramide by dihydroceramide desaturase (DES) results in ceramide, which can be glycosylated by glucosylceramide synthase (GCS) to form glucosylceramide or, alternatively, it can form galactosylceramide by the action of galactosyltransferase (CGT) or it can acquire a phosphocholine headgroup by sphingomyelin synthases (SMS) to eventually form sphingomyelin. The phosphorylation of ceramide by ceramide kinase (CK) results in ceramide 1-phosphate (C1P) (54–56).

**Figure 2:**
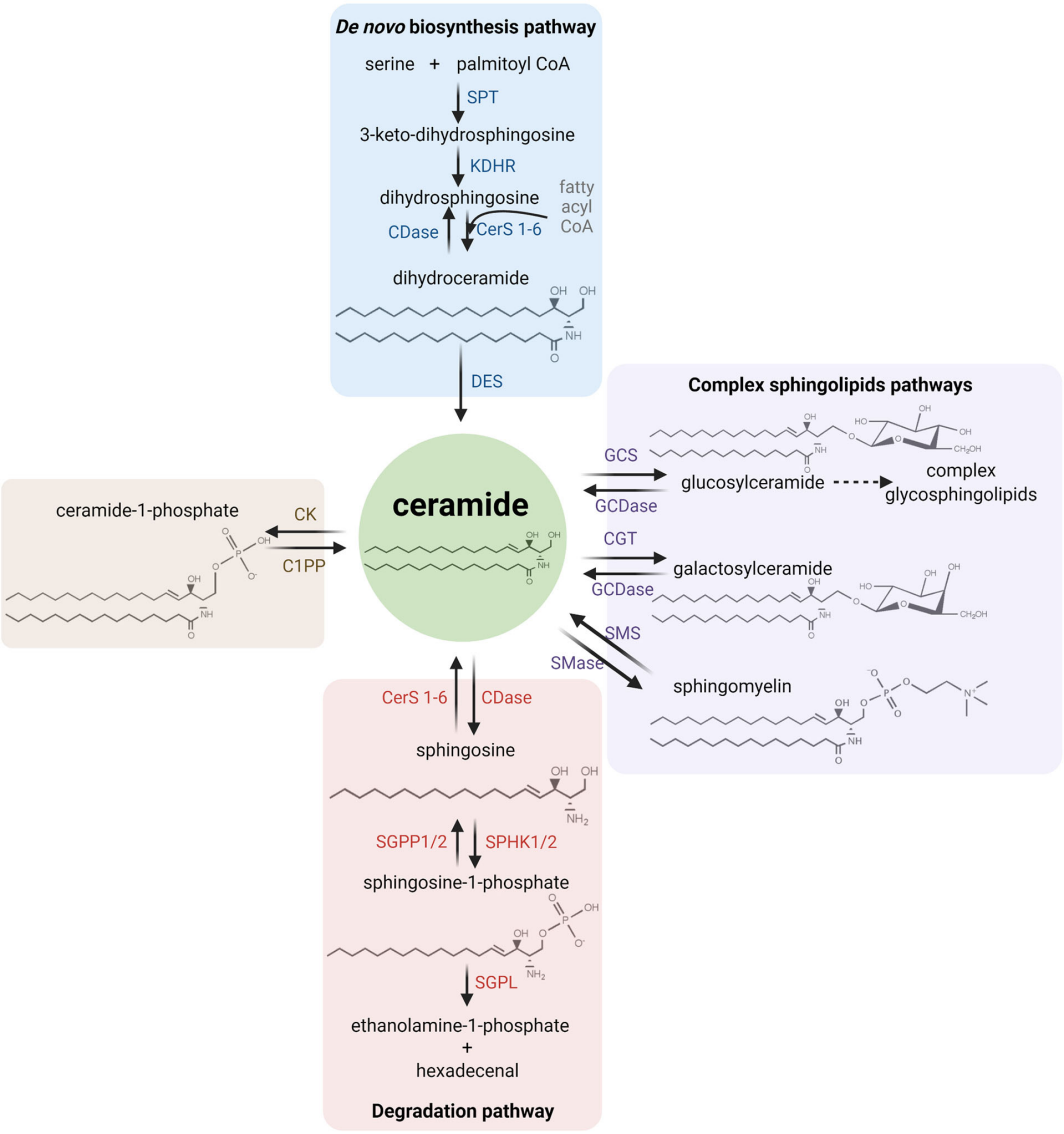
Sphingolipid metabolism. The synthesis of ceramide starts with the condensation of serine and palmitoyl CoA (the active form of palmitate) by serine palmitoyltransferase (SPT), resulting in 3-keto-dihydrosphingosine (also called 3-ketosphinganine). Subsequently, dihydrosphingosine (or sphinganine) is formed after the reduction of 3-keto-dihydrosphingosine by 3-ketosphinganine reductase (KDSR). Next, dihydrosphingosine is acetylated by a (dihydro-)ceramide synthase (referred to simply as ceramide synthase, CerS 1-6) to form dihydroceramide. Finally, desaturation of dihydroceramide by dihydroceramide desaturase (DES) results in ceramide. Ceramide can then be glycosylated by glucosylceramide synthase (GCS) to form glucosylceramide, or it can also form galactosylceramide by galactosyltransferase (CGT). On the other hand, ceramide can also acquire a phosphocholine headgroup due to the action of the sphingomyelin synthases (SMS) to eventually form sphingomyelin. Alternatively, ceramide kinase (CK) catalyzes the phosphorylation of ceramide to form ceramide 1-phosphate (C1P). Ceramidase (CDase) breaks down ceramide and generates sphingosine, which can be recycled back into ceramide by CerS 1-6 or phosphorylated by a sphingosine kinase (SPHK1/2) to form sphingosine-1-phosphate (S1P). In addition, S1P can be dephosphorylated by sphingosine phosphatase (SGPP1/2) to generate sphingosine again or it is cleaved by S1P lyase (SGPL) to obtain ethanolamine-1-phosphate and hexadecenal. On the other hand, the breakdown of sphingomyelin by sphingomyelinase (SMase), the breakdown of glucosyl/galactosylceramide by glucosyl/galactosylceramidase (GCDase) and the dephosphorylation of C1P by ceramide 1-phosphatase (C1PP) generate ceramide.

The catalysis of ceramide is performed by specific hydrolases. Ceramidase (CDase) breaks down ceramide and generates sphingosine, which can be recycled back into ceramide by CerS 1-6 or phosphorylated by a sphingosine kinase (SPHK1/2) to form S1P. Moreover, S1P can generate sphingosine by sphingosine phosphatase 1/2 (SGPP1/2) or it can exit the sphingolipids metabolic pathway through S1P cleavage by SGPL, obtaining ethanolamine-1-phosphate and hexadecenal. In addition, other hydrolases can produce ceramide, such as sphingomyelinase (SMase) which breaks down sphingomyelin (54). **Figure 2** illustrates the main metabolic pathways of sphingolipids metabolism.

## 4 Enzymes Involved in Sphingolipids’ Metabolism and Drug Resistance

### 4.1 Glucosylceramide Synthase (GCS)

GCS, encoded by the *UGCG* (*UDP-glucose ceramide glucosyltransferase*) gene, is the enzyme that transfers an UDP-glucose molecule to ceramide thus generating glucosylceramide, the precursor for all complex glycosphingolipids (54). Glycosylated sphingolipids cluster in the plasma membrane forming glycosphingolipid-enriched microdomains (GEMs), which are functional clusters that membrane proteins use as signaling platforms (57). Besides the plasma membrane, these GEMs can be found in the membranes of some subcellular organelles, such as mitochondria and are involved in the regulation of diverse cell functions including apoptosis (58).

Overexpression of GCS was reported in various cancers, such as breast and colon cancer (7). Moreover, drug resistant cancer cells from ovarian cancer, cervical cancer, melanoma, colon cancer and leukemia have shown GCS overexpression (11, 13, 59). Likewise, while doxorubicin exerts its action as an intercalating DNA agent and generates free radicals to damage growing cells, it also upregulates GCS expression and leads to drug resistance in cells through the modulation of the Sp1 transcription factor (15). Hence, efforts are being made to find a GCS inhibitor to downregulate GCS expression or inhibit its catalytic function.

#### 4.1.1 Breast Cancer

GCS overexpression is related to increased cellular proliferation (18) and poor prognosis in breast cancer patients (12). The most interesting feature of this enzyme is its functional connection with the *ATP binding cassette subfamily B member 1 (ABCB1)* gene, which encodes the ABCB1 protein, also called P-glycoprotein 1 (P-gp). Silencing of the *UGCG* gene by the promoter CpG island methylation shows an inverse correlation with drug resistance in ductal breast cancer cells (60). This means that the demethylation of the CpG island in the *UGCG* promoter could increase the generation of multidrug-resistant clones.

**Figure 3:**
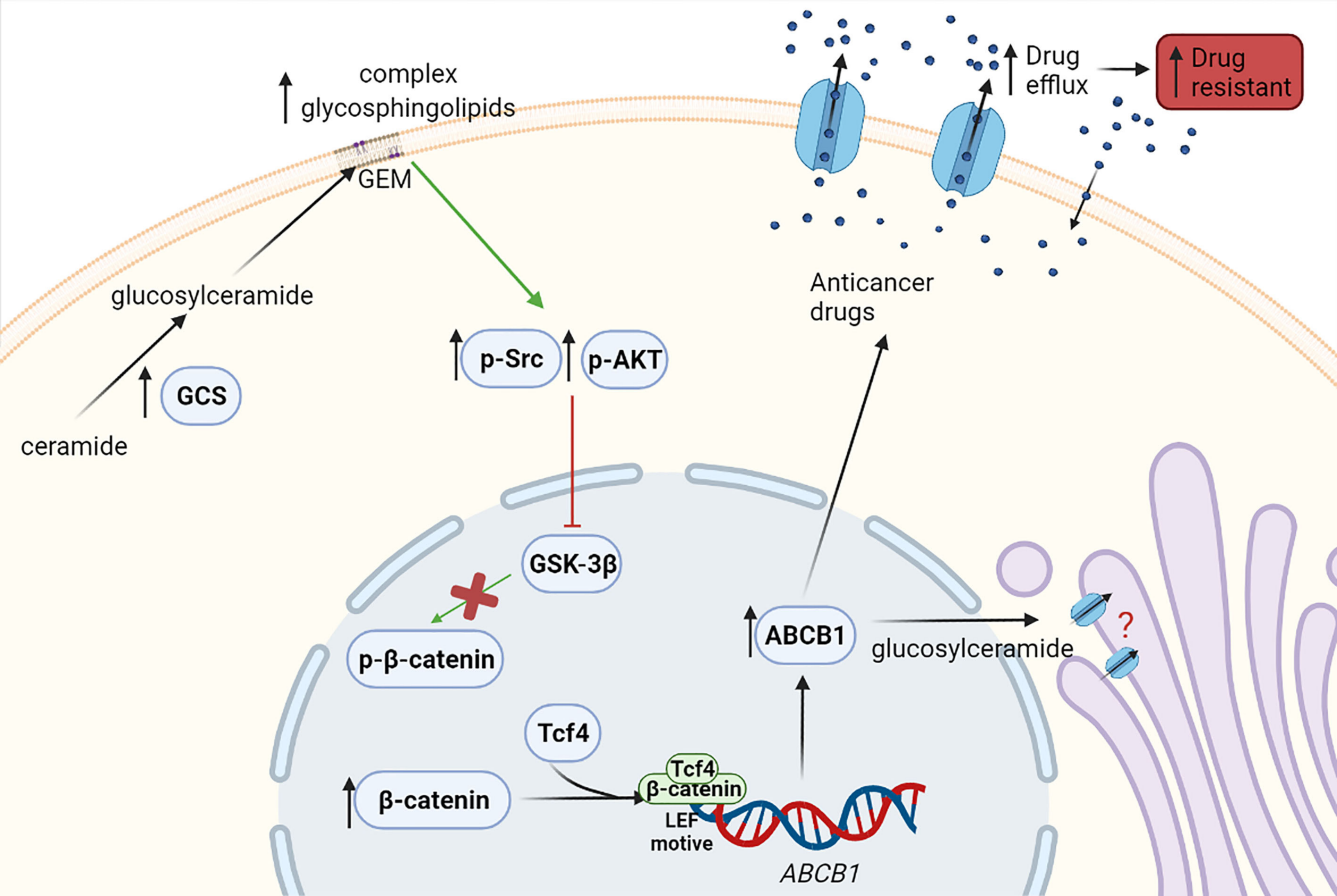
Effect of the enzyme GCS in drug resistance. Overexpressed GCS leads to increased concentrations of glycosphingolipids in the membrane, which may mediate the phosphorylation of Src (p-Src) and AKT (p-AKT) and their activation. Then, GSK-3β phosphorylated by p-Src or p-AKT becomes inactive and cannot phosphorylate β-catenin. Consequently, β-catenin cannot exit the nucleus and thus, β-catenin levels in the nucleus increase. β-catenin within the nucleus might bind in a transcriptional complex with Tcf4 (T-cell factor 4). This β-catenin/Tcf4 complex would then be able to bind a LEF (lymphoid enhancer factor) motive in ABCB1 (ATP-binding cassette subfamily B member 1) gene promoter, resulting in its transcription and increasing ABCB1 expression, thus leading to anticancer drug efflux and multidrug resistance. Likewise, it has been described that ABCB1 is located in the Golgi membrane and promotes the transfer of glucosylceramide from the cytosol to the Golgi, helping with the ceramide clearance from cytosol. However, further studies are required to validate this ABCB1 function. Green arrows show induction of expression and red arrows show inhibition.

Liu et al. demonstrated that GCS upregulates ABCB1 expression and modulates cancer drug resistance (59). In addition, studies from Zhang et al. revealed that in turn, ABCB1 can regulate GCS expression (61). Liu et al. also showed that silencing of GCS downregulates ABCB1 expression and sensitizes multidrug-resistant cells to chemotherapy through Src and β-catenin signaling (59). There is also evidence that GCS overexpression in breast cancer leads to AKT activation (p-AKT), which induces ABCB1 expression. At present, p-AKT is known to phosphorylate GSK-3β, the enzyme that phosphorylates β-catenin so it can exit the nucleus, hence this finding could validate the previous theory (18). Morad and Cabot suggested that ABCB1 is also located in the Golgi membrane acting as a flippase, and it is responsible for the transfer of glucosylceramide from the cytosol to the Golgi lumen, therefore promoting ceramide clearance (62). However, a different study revealed that flippase activity of ABCB1 is only needed for neutral, and not acidic, glycosphingolipids generation. There are also other studies which postulate that glucosylceramides regulate their own entrance to the Golgi apparatus, depending on the length of their ceramides’ chains, hence the flippase activity of ABCB1 would not be necessary or it would be an alternative mechanism to ceramide regulation (63). Further studies in this field are required to decipher the role of ABCB1 in the Golgi membrane and its connection with GCS (8) (**Figure 3**).

Although, it has been demonstrated that the GCS role in drug resistance is usually through ABCB1 overexpression, Liu et al. described that GCS is overexpressed in adriamycin-resistant MCF-7 breast cancer cells and that the multidrug resistance in these cells is independent from ABCB1 (59). Thus, it is possible that multidrug resistance in some cancer models is also derived from other unidentified proteins that are able to interact with GCS (9).

#### 4.1.2 GCS in Other Cancer Types

Inhibition of GCS in doxorubicin-resistant cells and temozolomide/paclitaxel-resistant cells is sensitized to treatment in colon cancer cells (64) and glioblastoma cells (10), respectively. In chronic myeloid leukemia (CML) cells, GCS can increase the expression of ABCB1 through NF-κβ signaling, a different mechanism than the one described in breast cancer (65). Moreover, in a murine melanoma model, inhibition of GCS in intrinsically chemoresistant cancer stem cells sensitized them to some genotoxic drugs (66).

### 4.2 Sphingosine Kinase (SPHK)

SPHK is a conserved family of lipid kinases and the enzymes responsible for the phosphorylation of sphingosine to generate S1P, maintaining the ceramide-S1P rheostat (67). There are two mammalian SPHK isoenzymes: SPHK1 and SPHK2; which have different locations in the cell and have different substrate specificities, kinetic properties and tissue expression (14, 16, 17). Regarding their structure, both isoenzymes have five conserved domains of ~50% identity (19). Accordingly, it is thought that some of the differences in the physiological effects between the two enzymes are due to the fact that SPHK2 has an extended NH_2_ terminus where a putative BH3 binding domain is located (68). Upon the first apoptosis stimulus, the BH3 domain is responsible to orchestrate apoptosis. The subcellular location of these isoenzymes is variable since it depends on the pathological state and tissue type. Usually, SPHK1 is found in the cell cytoplasm, whereas SPHK2 can be found either in the nucleus or in the cytoplasm (67, 69). Although only these two isoforms have been identified in human tissues, some studies suggest the presence of additional uncharacterized isoforms (67, 70). SPHK plays a pivotal role by regulating cell growth and acting as an oncogene in tumorigenesis (71–74). Furthermore, it has been shown that both isoenzymes exert important functions in angiogenesis, a crucial step in the metastatic spread process (16).

SPHK mRNA is overexpressed in some cancer tissues, including breast, colon, lung, ovary, stomach, uterus and kidney (75). In addition, the SPHK protein is overexpressed in prostate cancer and glioblastoma (76, 77). A study in the model organism *Dictyostelium discoideum* (a eukaryote species that belongs to the phylum Amoebozoa) showed that SPHK activity regulated the sensitivity to cisplatin, but not doxorubicin, etoposide and 5-fluoruracil, indicating the anti-cancer drug specificity. Overexpression of SPHKs sgkA and sgkB (homologous to human SPHK1, SPHK2, respectively) was observed to result in increased resistance to cisplatin (78). Similarly, it is expected that both SPHK1 and SPHK2 would provide cancer resistance in humans. Some examples based on cancer types are shown in the following sections.

#### 4.2.1 Prostate Cancer

Chemoresistant prostate cancer cells have high levels of SPHK1 (76). Pchejetski et al. proposed that SPHK1 activity is a chemotherapy sensor, since prostate cancer cells sensitive to the chemotherapeutic drug camptothecin reduced SPHK1 levels after treatment (20). In contrast, cells resistant to camptothecin did not significantly change SPHK1 levels after the treatment (20). Supporting this finding, Akao et al. demonstrated that SPHK1 activity in chemoresistant prostate cancer cells was significantly increased by treatment with camptothecin in a concentration-dependent manner and that this increment of the activity was due to increased protein and mRNA levels (76). Likewise, Pchejetski et al. also revealed that the inhibition of SPHK1 sensitized prostate cells not only to camptothecin but also to docetaxel (20). Since then, other studies have shown the sensitization to docetaxel by SPHK1 inhibition in docetaxel-resistant prostate cancer cells (22, 24). For example, Alshaker et al. showed that the SPHK inhibitor RAD001 sensitizes docetaxel-resistant cells to the docetaxel treatment both *in vitro* and *in vivo* (22). Additional *in vivo* studies are needed to demonstrate the potential of SPHK inhibitors in prostate cancer.

#### 4.2.2 Breast Cancer

It has been demonstrated that SPHK increases proliferation and survival in estrogen receptor (ER)-positive breast cancers, and it is associated with poor prognosis in the ER-negative subtype (25, 27). Moreover, overexpression of SPHK in MCF-7 cell line (ER-positive) promotes resistance to hormone therapy. A remarkable finding is that SPHK1 and SPHK2 expression among the different breast cancer subtypes is highly variable, making it difficult to generalize about the implication of these enzymes in breast cancer (69).

On the other hand, doxorubicin-resistant breast cancer cell lines showed a high expression of SPHK1 and its inhibition with fingolimod (immunosuppressive drug) caused a decrease in proliferation (21). Antoon et al. disclosed that ER-negative resistant breast cancer cells overexpressed SPHK; and specific inhibition of SPHK2 by ABC294640 decreased the growth of chemoresistant breast cancer cells *in vivo* enhancing doxorubicin and etoposide-induced apoptosis when using combined therapy (79). Some of these inhibitors are currently under clinical trials as described below.

#### 4.2.3 Glioblastoma

SPHK has been found overexpressed in glioblastoma cells and its overexpression has been correlated with poor prognosis (77). Several studies illustrated that the inhibition of SPHK1 results in a decrease in cell viability following the temozolomide treatment in temozolomide-resistant glioblastoma cells (23, 80, 81). Furthermore, there is also data that supports the therapeutic value of SPHK inhibitors as radiosensitizers (26).

#### 4.2.4 Chronic Myeloid Leukemia

Interestingly, resistance to tyrosine kinase inhibitors (such as imatinib) used for the treatment of chronic myeloid leukemia (CML) has been linked to alterations of sphingolipid metabolism and signaling. Overexpression of SPHK1 has been described to mediate imatinib resistance in CML patient-derived cells (82). The mechanism by which SPHK1 mediates imatinib resistance in CML cells is thought to take place through the modulation of the protein phosphatase 2 (PP2A). Inhibition of PP2A by SPHK1 might attenuate ubiquitination and proteasomal degradation of BCR-ABL, the mutated tyrosine kinase, enhancing its stability and resulting in subsequent drug resistance (83). Other studies revealed that SPHK1 overexpression in CML imatinib-resistant cells is regulated by signaling through PI3K, AKT2 and mTOR, with AKT playing a major role in the modulation of this resistance (84). Recently, Sun and Wang showed that a combined therapy of SPHK inhibitors and all-trans retinoic acid (ATRA), which has been described as limited to a treatment for CML, exert synergistic effects to inhibit proliferation in CML cells (85). Thus, the study of SPHK inhibitors might be important for CML treatment.

### 4.3 Acid Ceramidase (AC)

Ceramidases (CDases) are the enzymes responsible for the breakdown of ceramide to generate sphingosine. Human CDases can be classified into 5 different types, depending on their cellular location, primary structure and optimal pH needed for their catalytic activity: the 3 main ones are alkaline, neutral and acid CDases (86, 87). In particular, acid CDase (AC), encoded by the *ASAH1* gene, is a 50 kDa enzyme which belongs to the N-terminal nucleophile superfamily of hydrolases. AC is the only CDase that requires an additional protein to reach its optimal activity, saposin-D, a lysosomal protein which helps to present ceramide as a substrate to AC (88).

AC is overexpressed in some human cancers. For example, overexpression of AC has been demonstrated in melanoma (89) and prostate cancer (28) cell lines and biopsies as well as in head and neck squamous cell carcinoma (HNSCC) (90, 91), glioblastoma (92) and acute myeloid leukemia (AML) (93). Since AC upregulation has been linked to apoptosis resistance (29, 94), it is expected that the drugs able to decrease AC would be an efficient tool to sensitize resistant cells. The AC characterization is accordingly shown for the following cancer models.

#### 4.3.1 Melanoma

The most abundant skin cells (fibroblasts and keratinocytes) express low levels of AC. However, the number of cells with high levels of AC increase during cancer progression, as melanocytes start growing uncontrollably (89). The phenotype-switching model is a model of tumor progression that describes cancer development, resistance to therapy and metastasis; it is considered as one of the origins of intratumoral heterogeneity, a feature highly associated with therapy resistance. It states the existence of two different phenotypes: the proliferative phenotype, that is less invasive, and the invasive phenotype, which is less proliferative (30, 31, 95). Studies in melanoma cells revealed that lysosomal AC and the sphingolipid metabolism drive the transition between the proliferative and the invasive phenotype (96). AC expression is higher in proliferative melanoma cells compared with other skin cells (89). Interestingly, in metastatic melanoma, it has been described that downregulation of AC, but not neutral or alkaline CDases, increases ceramide levels in the cell and confers the cells sensitization to dacarbazine (97), the main chemotherapeutic drug (DNA-alkylating agent) used for this type of cancer before immune checkpoint inhibitors or BRAF inhibitors became available for clinical use (98). Recently, it has been reported that AC ablation restores melanoma sensitivity to doxorubicin, a different chemotherapeutic agent used for melanoma treatment that affects sphingolipids’ metabolism, since it forces cells treated with doxorubicin to undergo apoptosis. It is thought that this restoration of doxorubicin sensitivity is due to the increase of ceramide accumulation subsequent to AC inhibition (99). Overall, AC inhibition or downregulation could represent an interesting approach to sensitize melanoma cells to some cancer drugs.

#### 4.3.2 Prostate Cancer

It has been shown that AC is overexpressed in 60% of prostate cancer tumors (28). This overexpression is linked to prostate cancer progression, and it modulates sphingolipid levels in prostate cancer cells, resulting in higher levels of very long chain ceramides. In addition, higher levels of AC made the cells more resistant to apoptosis following treatment with doxorubicin, etoposide, cisplatin or gemcitabine, known chemotherapeutics used to treat prostate cancer. Accordingly, downregulation of AC reversed the resistance to these therapies. For this reason, the combination of current chemotherapy and AC inhibitors is proposed as an efficient way to improve prostate cancer treatment (94). In this sense, Kus et al. demonstrated the induction of apoptosis in prostate cancer cells by using a ceramidase inhibitor, ceranib-2 (100). Besides its role in chemotherapy resistance, AC overexpression has also been linked to resistance to radiotherapy in prostate cancer (101, 102). This is important because resistance to radiotherapy is an undeveloped field of exploration and in the case of prostate cancer - in which it is relatively easy for cells to acquire resistance to androgens – the treatment with radiotherapy acquires an essential role.

#### 4.3.3 AC in Other Cancer Types

The implication of AC in treatment resistance in breast cancer, AML, HNSCC and glioblastoma has also been explored. In glioblastoma, it has been demonstrated that AC levels are higher in radioresistant tumors, suggesting that AC may confer radio resistance (103). In this particular type of cancer, AC has been directly linked to the increase in the survival of glioblastoma stem-like cells (GSCs), that are usually more resistant to anticancer therapies (92). In HNSCC, it has been shown in both *in vitro* and *in vivo* techniques that the use of an AC inhibitor (LCL 204) sensitizes the tumor to FasL gene therapy. For this reason, the combination of FasL gene therapy with LCL 204 may become an effective new treatment for HNSCC tumors (90). With a proteomics/bioinformatics approach, Yang et al. found that AC, among other proteins, was associated with breast cancer drug resistance (32, 39). Finally, inhibition of AC in AML has been shown to increase ceramide levels and induce apoptosis (93, 104). Hence, AC can be considered as a potential target for several cancer models of a particularly aggressive nature such as HNSCC, melanoma or glioblastoma therapy.

### 4.4 Sphingomyelinases (SMases)

SMases are the enzymes that carry out the hydrolysis of sphingomyelin (SM) to generate ceramide. In 1999, Samet and Barenholz (33) proposed a classification of eukaryote SMases into 5 categories, which differed based on cation dependency, pH cation optima and intracellular location: acid sphingomyelinase (aSMase), secretory sphingomyelinase (sSMase), $\text{M}{\text{g}}_{2}^{+}$ -dependent neutral sphingomyelinases (nSMase), $\text{M}{\text{g}}_{2}^{+}$ -independent neutral sphingomyelinases and alkaline sphingomyelinases (bSMases) (33, 105, 106). At present, they are usually labelled by their optimal pH (54): aSMases, located in the lysosome and lipid rafts; nSMases, located in the plasma membrane; and bSMases, located in the endoplasmic reticulum (34). Most SMase studies focus on aSMase, encoded by the *SMPD1* (sphingomyelin phosphodiesterase 1) gene (35), which produces a protein with different molecular weights (from 58kDa to 75kDa), depending on the tissue of origin (36, 105). It has been estimated that around 70% of all cellular aSMase are located in lipid rafts, which constitute an important group of the plasma membranes’ structures involved in the regulation of various cellular processes. Specifically, aSMase could be involved in the increase of membrane fluidity because its activity results in cholesterol release from the membranes (37).

The action of SMases has been found to be an essential step for the efficacy of chemotherapy and radiotherapy (38, 40, 41). For example, Santana et al. discovered that aSMase-deficient human lymphoblasts and mice specimens cannot induce apoptosis after ionizing radiation treatment (42). Moreover, many studies have linked this enzyme with drug resistance in glioblastoma (107), melanoma (43), colon cancer (44), ovarian cancer (108) and non-small cell lung cancer (NSCLC) (109). Thus, the potential role of this enzyme not only in chemo- but radio resistance might situate aSMase as a central axis in therapy resistance. These studies are summarized in the next sections. The role of SMases in different cancer models is shown below.

#### 4.4.1 Glioblastoma

**Figure 4:**
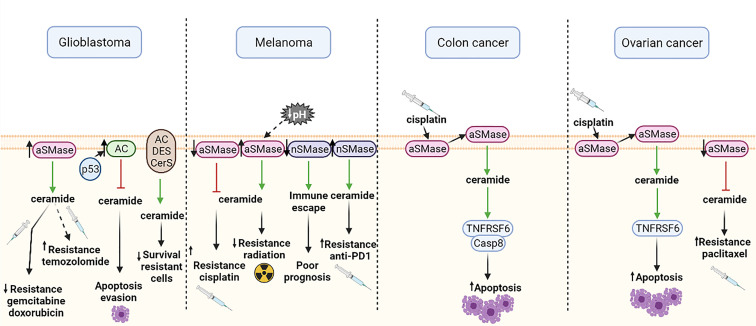
Effects of different SMase expressions in chemotherapy response or cellular functions in glioblastoma, melanoma, colon cancer and ovarian cancer. Green arrows show induction of expression and red arrows show inhibition. aSMase, acid sphingomyelinase; nSMase, neutral sphingomyelinase; AC, acid ceramidase; DES, dihydroceramide desaturase; CerS, ceramide synthase; Casp8, caspase 8; TNFRSF6, tumor necrosis factor receptor superfamily member 6. All the figures were created with BioRender.

In glioblastoma, some studies showed that aSMase overexpression sensitized glioma cells to gemcitabine and doxorubicin, two chemotherapeutics used for glioblastoma treatment (107). Paradoxically, Gramatzki et al. detected opposite results several years later with a different chemotherapeutic, demonstrating that the overexpression of aSMase did not sensitize glioblastoma cells to radiation or chemotherapy with temozolomide, the current chemotherapeutic considered as standard (110). Nevertheless, Gramatzki et al. also found in the same study that increased levels of ceramide by aSMase-independent pathways decreased the survival of temozolomide-resistant glioma cell lines (110). A different study showed that aSMase, but not nSMase, hydrolyzed sphingomyelin to generate ceramide and induced apoptosis in p53-deficient glioblastoma cells; while in p53 wild-type glioblastoma cells, p53 expression upregulated AC and blocked the ceramide response, thus allowing the cells to evade apoptosis. Therefore, p53 status might be important for the response to treatment in glioma cells (111). The exact mechanism of p53-ceramide interaction is still not fully understood, but it is known that SMases can regulate apoptosis in glioma cells with differential responses, somehow related to p53 expression and p53 downstream targets (112). **Figure 4** represents how the SMase expression modulates the response to chemotherapy.

#### 4.4.2 Melanoma

Studies of the role of SMase in melanoma cells show that cells with low aSMase expression exhibit higher resistance to cisplatin, probably because of their lower levels of ceramide (43). Furthermore, aSMase expression is also linked to radiotherapy resistance, since overexpression of aSMase in mice with melanoma sensitized the tumors to irradiation. Regarding this matter, some studies suggest that a lower pH of some solid tumors may increase the activity of aSMase and, consequently, radiosensitivity (113). Likewise, it has been demonstrated that downregulation of nSMase2 contributes to immune escape and is associated with poor prognosis in human melanoma. Recently, it has been suggested that nSMase2 overexpression might be useful to overcome resistance to anti-PD-1 (114) (**Figure 4**). This has clinical relevance since immunotherapy has been revealed as an effective alternative therapy in approximately 20% of metastatic melanoma patients.

#### 4.4.3 Colon Cancer

As mentioned earlier, aSMase is mainly located in the plasma membrane lipid rafts. Lacour et al. proposed a molecular mechanism of cisplatin-induced cytotoxicity involving aSMase in human colon cancer cells (44). This study suggested that treatment with cisplatin induces a translocation of aSMase to the extracellular surface of the plasma membrane. Subsequently, ceramide production is activated and the TNFRSF6 (tumor necrosis factor receptor superfamily member 6) proapoptotic protein and caspase 8 (Casp8) are redistributed into membrane fractions enriched in cholesterol and sphingolipids (44) (**Figure 4**). However, the detailed molecular mechanism of cisplatin-induced cytotoxicity and how aSMase is involved still remains unclear. Further investigation in this field is required to unveil the specific molecular mechanism in order to design a personalized therapy.

#### 4.4.4 Ovarian Cancer

Maurmann et al. postulate that aSMase activation and increased TNFRSF6 levels in cisplatin-resistant ovarian cells may suggest a similar mechanism as the one described by Lacour et al. for cisplatin-induced cytotoxicity (44, 108). In this study, they also demonstrated that in cisplatin-resistant cells, the aSMase activation of TNFRSF6 is dependent on cisplatin concentration (108). Furthermore, besides the cisplatin resistance, it has also been described that aSMase inhibition in ovarian cancer cells increase paclitaxel treatment resistance. It has been reported that aSMases and nSMases are activated by paclitaxel treatment in drug-sensitive cells, but are not affected in resistant cells (115) (**Figure 4**). Hence for this cancer model, alternative therapies should cover the targeting of resistant cells.

#### 4.4.5 SMase in Other Cancer Types

There is also evidence of the importance of aSMase in other types of cancer, such as NSCLC, but further studies are needed to decipher the role of this enzyme in chemoresistance (109).

## 5 Clinical Implications

Sphingolipids act both as structural components of membranes and as bio-effector molecules. In the last decade, the study of sphingolipids has been important for the development of innovative therapeutic strategies for drug-resistant tumors. The central axis of sphingolipids´ metabolism is ceramide, which has been the focus of numerous studies. Ceramide can be generated or catabolized by many enzymes, leading to different sphingolipids. AC breaks down ceramide to produce sphingosine, and its inhibition usually sensitizes drug-resistant cells to treatment. Similarly, the inhibition of SPHK, that phosphorylates sphingosine into S1P, and GCS, that generates glucosylceramide from ceramide, also sensitizes drug-resistant cells. All these enzymes are involved in the elimination of ceramide by the cell; hence, lower levels of ceramide are linked to cancer drug resistance. In contrast, the inhibition of SMases, which generate ceramide from the sphingomyelin breakdown, leads to increased drug-resistance in cancer. Taking all of these factors into account, it seems clear that the regulation of these enzymes affects the levels of ceramide in the cell, subsequently providing resistance or sensitization to some chemotherapeutics (15). Many authors support the idea that, independently of which enzyme is deregulated, drug resistance is acquired when there are low levels of ceramide in the cell. For example, doxorubicin enhances ceramide production inside the cell by activation of SMases or enzymes of ceramide synthesis, which should drive the cell to apoptosis (15, 116). However, it has been demonstrated that ceramide upregulates GCS expression, resulting in a resistant phenotype (15). Unlike the other three enzymes (AC, SMase and SPHK), the drug resistance produced by GCS may not only be related to ceramide generation, since GCS products (glycosphingolipids) are involved in the increase of multi-drug resistance through ABCB1 expression. Moreover. GCS expression which is epigenetically regulated correlates with drug resistance in breast cancer cells (60). Nevertheless, to our knowledge the mechanism has not been identified by which SMase, SPHK or AC modulate their expression levels.

In general, although targeting sphingolipids such as ceramide or glycosphingolipids is a major approach to deal with drug resistance in cancer, targeting crucial enzymes involved in these pathways seems to be a promising strategy in this field. These enzymes could be considered as potential targets to modulate ceramide levels in the cell and, therefore, resistance to treatment, particularly in drug-resistant tumors. Further research about the protein levels of these enzymes should be extended to all cancer types to determine if the use of the modulators of these enzymes (inhibitors or enhancers) would be effective either to prevent or to revert resistance to current conventional treatments. Overall, inhibitors of AC, GCS and SPHK and enhancers of SMases could be potential targets for the treatment of drug-resistant tumors in the indicated cancer types. Some modulators for these enzymes have already been studied and are currently used for clinical purposes to manage treatment resistance. Unfortunately, none of these modulators has been approved for cancer treatment to date, although some of them are currently under study. For instance, tamoxifen, a classical drug to treat several cancer types with a complex action mechanism, inhibits AC (117). Therefore, for those cancers that are not treated with tamoxifen, administering this drug in combination with the standard treatment may prevent resistance to that standard treatment (62, 117). Likewise, treatment with doxorubicin or etoposide leads to a significant downregulation of SPHK1 and, consequently, an accumulation of ceramide within the cell which confers therapy sensitization to resistant cells (118).

In addition, some sphingolipid-targeting drugs are under study because of their potential use in cancer treatment in chemoresistant tumors. This is the case of the SPHK1 inhibitor PF-543 since it has been demonstrated that its dansylated form (DPF-543) provides high cytotoxicity. This compound also triggers aSMase activation, leading to a higher accumulation of ceramides within the cell (119). Other SPHK inhibitors, such as F-12509, SKI-I and FTY720 (Fingolimod), specifically inhibit SPHK1; ABC294640 (Opaganib) and K145 specifically inhibit SPHK2; and SKI-II which inhibits both SPHK1 and SPHK2. The inhibitor ABC294640 has recently reached phase 2 of clinical trials as a drug for refractory multiple myeloma patients (NCT02757326, www.clinicaltrials.gov). FTY720 is also being tested in clinical trials (www.clinicaltrials.gov) for breast cancer (NCT02490930) and glioma patients (NCT03941743). Likewise, several AC inhibitors, such as LCL521 and ceranib-2, have been described to improve chemotherapy effects (120, 121). Additional research on these molecules is required to evaluate their potential effects as a cancer therapy. Similarly, some GCS inhibitors, such as PPMP (D,L-Threo-1-phenyl-2-palmitoylamino-3-morpholino-1-propanol) and PDMP (D,L-Threo-1-phenyl-2-decanoylamino-3-morpholino-1-propanol), have been tested *in vitro* with promising results. For instance, PPMP increased sensitivity of chemoresistant HNSCC to cisplatin and PDMP increased sensitivity of pancreatic cells (122, 123).

Although these compounds reached clinical trials, most of them did not show sufficient efficacy in patients to continue the study. The lack of efficacy of these compounds in patients could be explained by several reasons. First, different enzymes of sphingolipid metabolism are thought to have redundant functions, meaning that even if one enzyme is inhibited by one drug, a different enzyme could perform its function. For example, some studies suggest that SPHK1 and SPHK2 may have redundant functions and that one may compensate for the deficiency of the other (16, 17). A second explanation could be that these compounds that reached clinical trials present non-specific effects or that the compound is effective at high doses that are not clinically tolerable in patients (liver toxicity). A third reason could be the adverse effects that such compounds cause in patients under study, leading to discontinuation and definitive transfer to the clinic. Adverse effects may include hematuria, vomiting, hyperglycemia, personality disorders, fever, and dizziness. Overall, these compounds remain a promising strategy to treat chemoresistant tumors once these problems are overcome.

## 6 Conclusions

1. Reliable markers based upon sphingolipid enzymes able to stratify patients (i.e., responders to cancer therapy versus non-responders) into current therapies including immunotherapy are expected.
2. AC is a promising target and several AC inhibitors are already under study, although no clinical trials have been performed with these inhibitors to date.
3. Since SMases need to be overexpressed to potentially reduce drug resistance, such overexpression has only been reached by the addition of SMases or genetic modulation, but not pharmacologically. Additional studies of this enzyme are needed to test if the overexpression of this enzyme can be achieved pharmacologically.
4. Research on the characterization of SPHK inhibitors is growing due to the relation between GCS and multidrug resistance. However, none of those research projects has reached clinical trials yet. Further research on GCS and multidrug resistance relation in different cancer types is required, since the majority of the studies published to date have been performed in breast cancer.
5. The most promising drugs to modulate sphingolipids are SPHK inhibitors, which have been subject to in-depth study. Currently, some of these inhibitors, such as ABC294640 and fingolimod are in clinical trials with good results.
6. In the next few years, animal models should reveal whether the therapeutic exploitation of sphingolipids metabolism modulation will determine future treatments for oncology patients.

## Author Contributions

MB: writing the manuscript. AS-G: design of figures. YG-M: reading and improving the scientific quality. CM: reading and improving the scientific quality. IR: reading and improving the scientific quality. ML: supervision and correction of the manuscript. All authors contributed to the article and approved the submitted version.

## Conflict of Interest

The authors declare that the research was conducted in the absence of any commercial or financial relationships that could be construed as a potential conflict of interest.

## Publisher’s Note

All claims expressed in this article are solely those of the authors and do not necessarily represent those of their affiliated organizations, or those of the publisher, the editors and the reviewers. Any product that may be evaluated in this article, or claim that may be made by its manufacturer, is not guaranteed or endorsed by the publisher.
